# Annotations

**Published:** 1909-04-17

**Authors:** 


### ANNOTATIONS.

### Neurasthenia and Quackery.

We wonder how long it is to be possible for brazen unscrupulosity to foist on the public and the profession goods of very small value—almost none at all—at inflated prices. An especially gross case in point has recently been exposed in the weekly newspaper *Truth*. The "remedy" in question has been extensively advertised as a sovereign cure for the "twentieth century disease—neurasthenia," along with blood-curdling descriptions of the symptoms and the awful sequelæ of its uncombated ravages. One of the most amusing features of the campaign of mendacity by which the sale of this product—antineurasthin—has been pushed is the defeat of the Revenue authorities by the proprietors, who successfully maintained that their article is of the nature of a food, and not a drug; and consequently that it is not liable to pay stamp duty. The truth of this contention is clearly manifest when it is added that antineurasthin consists, according to an analysis conducted by the *British Medical Journal*, of dried egg and milk, and that the four tablets of it recommended as a daily dose are approximately equal to a teaspoonful of the former and two ounces of the latter. The comedy thickens when we learn that the eminent analyst, whose distinctions and qualifications take up seven or eight lines of print, lives in upstairs lodgings in a small house, and when not engaged in writing glowing testimonials of the healing virtues of patent remedies spends his time as a member of a travelling theatrical company.

### Improved Mortuaries.

Attention has many times been called by coroners and coroners' juries, and in the columns of the medical press, to the scandalously imperfect equipment of public mortuaries in Britain generally and in London especially. Since the system of holding inquests in hospitals, where up-to-date mortuaries are now usual, has been virtually abandoned, this backward state of the metropolis has been more than ever forced upon the attention of juries, pathologists, and, in fact, everyone whose duties call him to a coroner's inquest. Those more immediately concerned must almost have given up hope of seeing any remedy provided; but we are very glad to notice, in the report of an inquest held at Southwark, that the coroner (Dr. Waldo) was able to announce to the jury that the City Corporation has agreed to install the Rechter apparatus for the treatment of bodies deposited there. This consists in an air-tight chamber, wherein the body can be chemically treated in such a way as to prevent decomposition, and at the same time to place no obstacle in the way of subsequent post-mortem examination. The advantages of some such arrangement over the haphazard plan of allowing all corpses to undergo putrefaction without hindrance are varied. Thus unidentified persons can be preserved for some time pending identification; also the bodies of those in whom post-mortem changes are already extensive, as in those found drowned, are protected from such further progressive change as may hinder proper elucidation of medico-legal points connected with the mode of death and of identification. To pathologists and any who may have to undertake autopsies the value of the change is incalculable, from the point of view not only of their own convenience, but also, and more important, of their health and the efficient discharge of their duties. To the City Coroner congratulations may be offered on the success of his long campaign of reform, which will be a good omen to the other coroners of London who have also interested themselves in the question. The London County Council ought not to be outdone by the City Corporation, and it is to be hoped that steps in the right direction will soon be taken.

### The Government and the Alcohol Congress.

We have repeatedly expressed the opinion that the immense social problem of alcoholism can only be dealt with satisfactorily by means of organised investigations along scientific lines. We anticipate, therefore, that some definite progress in the combating of the evil will result from the twelfth International Congress on Alcoholism, which will be held at the Imperial Institute, London, on July 19 and following days; and we are glad to learn that the delegates will be entertained at a reception by the Government at the Imperial Institute on the evening of Monday, July 19. The arrangements will be made and the expenses defrayed by the Government, and, presumably as a guarantee of good faith, we note that only non-alcoholic drinks will be provided for the refreshment of the delegates. The Foreign Office has invited the Governments of the following countries to send official representatives to the congress:—The United States, France, Germany, Denmark, Norway, Sweden, Switzerland, Russia, Austria, Hungary, Roumania, Italy, Belgium, Holland, Uruguay, and Mexico. The Colonial Office has also extended similar invitations to the Governments of all our self-governing Colonies; and the Government of India is being consulted by the India Office with a view to the appointment of a representative for India. By permission of the Home Office, Dr. R. W. Branthwaite, the Inspector under the Inebriates Acts, will attend the Congress, as will Lieutenant-Colonel McHardy, chairman of the Prisons Commissioners for Scotland, and Sir George O'Farrell, Inspector of Lunatic Asylums in Ireland. It is estimated that the delegates will number nearly 2,000, of whom probably 500 will come from foreign countries and the Colonies. The Hon. President is the Duke of Connaught and the Chairman of Committee is the Dean of Hereford. We earnestly hope that a truly scientific spirit will animate the whole of the proceedings of the Congress, and that every speaker will remember that the cause of temperance in the present state of public opinion has more to fear from intemperate language on the part of its advocates than from almost anything else. With this word of caution we beg to offer our most cordial wishes for the success of the Congress.

